# Development and Validation of a Four Adenosine-to-Inosine RNA Editing Site-Relevant Prognostic Signature for Assessing Survival in Breast Cancer Patients

**DOI:** 10.3389/fonc.2022.861439

**Published:** 2022-04-12

**Authors:** Jian Wan, Shizhen Chen, Anqin Zhang, Yiting Liu, Yangyang Zhang, Qinghua Li, Ziqi Yu, Yuwei Wan, Lei Yang, Qi Wang

**Affiliations:** ^1^ The First Affiliated Hospital, Jinan University, Guangzhou, China; ^2^ Breast Disease Center, Guangdong Women and Children Hospital, Guangzhou, China; ^3^ The State Key Lab of Respiratory Disease, Institute of Public Health, Guangzhou Medical University, Guangzhou, China

**Keywords:** A-to-I RNA editing, breast cancer, nomogram, overall survival, disease-free survival

## Abstract

**Background:**

Adenosine-to-inosine RNA editing (ATIRE) is increasingly being used to characterize cancer. However, no studies have been conducted to identify an ATIRE signature for predicting cancer survival.

**Methods:**

Breast cancer (BRCA) samples with ATIRE profiles from The Cancer Genome Atlas were divided into training (n = 452) and internal validation cohorts (n = 311), and 197 additional BRCA patients were recruited as an external validation cohort. The ATIRE signature for BRCA overall survival (OS) and disease-free survival (DFS) were identified using forest algorithm analysis and experimentally verified by direct sequencing. An ATIRE-based risk score (AIRS) was established with these selected ATIRE sites. Significantly prognostic factors were incorporated to generate a nomogram that was evaluated using Harrell’s C-index and calibration plot for all cohorts.

**Results:**

Seven ATIRE sites were revealed to be associated with both BRCA OS and DFS, of which four sites were experimentally confirmed. Patients with high AIRS displayed a higher risk of death than those with low AIRS in the training (hazard ratio (HR) = 3.142, 95%CI = 1.932–5.111), internal validation (HR = 2.097, 95%CI = 1.123–3.914), and external validation cohorts (HR = 2.680, 95%CI = 1.000–7.194). A similar hazard effect of high AIRS on DFS was also observed. The nomogram yielded Harrell’s C-indexes of 0.816 (95%CI = 0.784–0.847), 0.742 (95%CI = 0.684–0.799), and 0.869 (95%CI = 0.835–0.902) for predicting OS and 0.767 (95%CI = 0.708–0.826), 0.684 (95%CI = 0.605–0.763), and 0.635 (95%CI = 0.566–0.705) for predicting DFS in the three cohorts.

**Conclusion:**

AIRS nomogram could help to predict OS and DFS of patients with BRCA.

## Introduction

Female breast cancer (BRCA) has now been the most commonly diagnosed cancer with an estimated 2.3 million new cases in 2020 worldwide (1). Over the past decades, the long-term survival rate of female BRCA has dramatically increased due to advances in early diagnosis and prompt treatment. The data from Surveillance, Epidemiology, and End Results (SEER) shows the 5-year survival rate of female BRCA approached 90.3% in the United States (https://seer.cancer.gov/statfacts/html/breast.html), while in China, the number was about 82.0% (2). However, the high incidence of female BRCA still caused an estimated 684,996 deaths in 2020 (1). Besides, improved treatments such as adjuvant systemic therapy result in inappropriate treatment in many patients, including both unnecessary treatment and insufficient curing. Therefore, it is of great value to develop a reliable prognostic model to identify patients at high risk of death or disease recurrence, which may suggest changes in life health management.

Accompanied by the revelation of transcriptomics in BRCA, various prognostic models have been developed to help physicians assess the survival of BRCA patients (3–10). These prediction models were mostly established by gene expression data recorded by The Cancer Genome Atlas (TCGA) database (https://portal.gdc.cancer.gov/) or Gene Expression Omnibus (GEO) database (https://www.ncbi.nlm.nih.gov/geo/). Nevertheless, all these models have limitations in clinical practice in terms of their veracity, reliability, and reproducibility, which are always at the mercy of mobility and detection instability in gene expression. A novel type of molecular markers may contribute to overcoming these challenges. RNA editing is a molecular process through which some cells can make discrete changes to specific nucleotide sequences within an RNA molecule (11). Compared to gene expression, RNA editing is more tumor-specific (12, 13) and irrespective of inter-individual variability at the amount of isolated RNA and reference gene selection. Therefore, as a biomarker, RNA editing is superior to RNA expression in terms of test stability and reliability.

More than 70% of RNA editing in humans is adenosine-to-inosine RNA editing (ATIRE) (14), which results in adenosine-to-inosine transitions at particular sites of pre-mRNA. In the process of post-transcription and translation, inosine is recognized as guanosine (G). Recent bioinformatics analyses have revealed transcriptome-wide ATIRE profiling and identified survival-related ATIRE sites in cancer by analyzing TCGA sequencing data (15, 16). However, no studies have validated both the reality and prognostic correlation of these sites in cancer. In this study, we aimed to develop and validate an ATIRE-based risk score (AIRS) for assessing the probability of overall survival (OS) and disease-free survival (DFS) in patients affected by BRCA, and for the first time, we constructed a novel prognostic model using ATIRE with considerable accuracy on predicting BRCA OS and DFS.

## Material and Methods

### Dataset Preparation and Studied Subjects

ATIRE profiling of TCGA-BRCA patients in cancer tissues was downloaded from the synapse (https://www.synapse.org/#!Synapse:syn4382382) (15). The corresponding clinicopathological parameters of indicated patients were downloaded from the cBioPortal (https://www.cbioportal.org/). Patients were excluded if they lacked data about ATIRE level, OS, and TNM stages. The remaining patients were randomly allocated to a training cohort (n = 452) and an internal validation cohort (n = 311). Besides, an external validation cohort (n = 197) was randomly recruited in Guangdong Women and Children’s Hospital and Health Institute from January 2014 to September 2019 in Guangzhou city. All cases were histopathologically confirmed and followed up by phone by one of us (JW). After a median follow-up of 68 months, 89.3% of patients (176/197) were followed up successfully and provided complete OS information. Meanwhile, 83.8% of patients provided DFS data. OS was defined as the length of time from the date of diagnosis for BRCA that patients diagnosed with the disease are still alive. DFS was defined as the time after surgery to relapse, second cancer, or all-cause death, whichever came first. There were no significant differences in frequency distributions of clinicopathological characteristics between the cases lost to follow-up and those to be analyzed (**Supplementary Table S1**). All individuals have signed written informed consent. The study was approved by the Institutional Review Board of Guangzhou medical university.

### Identification of Breast Cancer-Survival Related Adenosine-to-Inosine RNA Editing Sites and Generation of Adenosine-to-Inosine RNA Editing-Based Risk Score Model

**Figure 1:**
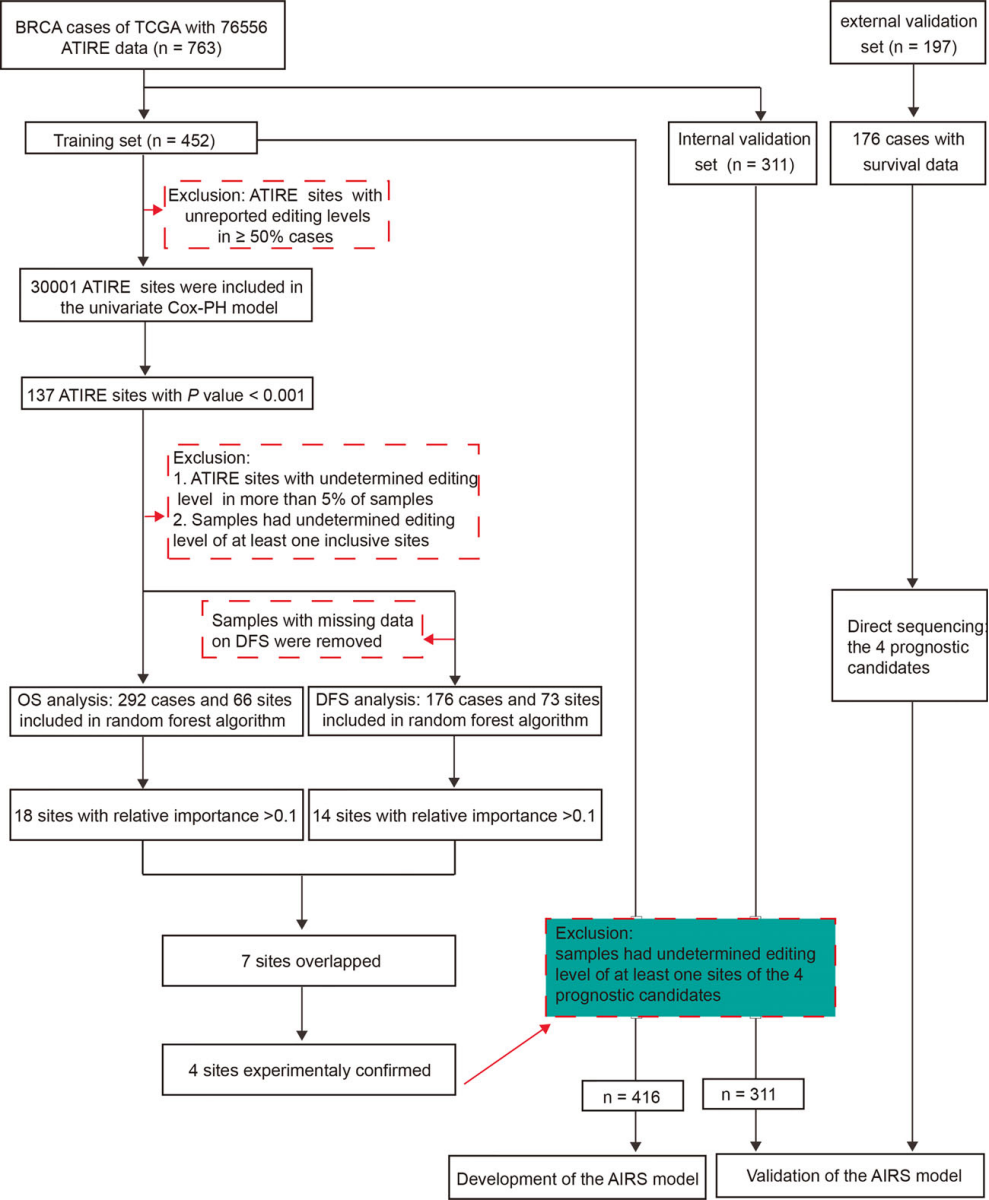
The workflow of generation of an AIRS model for BRCA OS and DFS. AIRS, adenosine-to-inosine RNA editing risk score; BRCA, breast cancer; OS, overall survival; DFS, disease-free survival.

**Figure 1** presents the flowchart of AIRS model construction for BRCA survival. After elimination of sites with low authenticity whose editing levels were not reported in more than half of the samples, 30,001 ATIRE sites were included in the univariate Cox proportional hazards (Cox-PH) model in the training cohort. Then the sites with *p* < 0.001 were submitted to the forest algorithm analysis, a non-parametric machine learning strategy that was recently used for building a risk prediction model in survival analysis (17). Due to the limitation of forest algorithm analysis, these sites with undetermined editing levels in more than 5% of samples and samples that had undetermined editing levels at any of the inclusive sites were removed. After the median split of samples into high and low editing groups according to the editing level, the common sites for OS and DFS with relative importance > 0.1 were preselected for the construction of the AIRS model.

To verify the reality of these preselected ATIRE sites, complementary DNA (cDNA) and genomic DNA (gDNA) were isolated from five randomly selected BRCA tissues and were used as templates for PCR. The PCR products were purified and then directly sequenced. The primers used are presented in **Supplementary Table S2**.

Empirically supported ATIRE sites were finally selected for AIRS model construction. To maximize the efficiency and robustness of the model, the editing level of each ATIRE was classified into low, medium, and high through the optimal grouping method using the X-Tile Software (https://medicine.yale.edu/lab/rimm/research/software/) (18). Cox-PH was used to measure the hazard ratio (HR) and 95% CI for the association between each ATIRE and BRCA survival. Then, an AIRS model was established by summing the effect of each site by its respective weight (lnHR).

### Validation of Adenosine-to-Inosine RNA Editing-Based Risk Score Model

Associations of the AIRS model with BRCA OS and DFS were validated in the internal and external validation cohorts. The PCR followed by sequencing was performed to determine the editing level of each selected ATIRE site in the external validation cohort. The editing level was calculated by the ratio between the height of peak corresponding to G and the sum of the height of peaks corresponding to G and A.

### Construction and Validation of an Adenosine-to-Inosine RNA Editing-Based Risk Score-Based Nomogram

A nomogram incorporating AIRS and clinicopathological features including age at diagnosis, TNM stages, estrogen receptor (ER), and progesterone receptor (PR) status was constructed by means of the “rms” package in R (version 4.0.1). To validate the nomogram, the total point of each patient in the two validation cohorts was calculated according to the established nomogram. The predictive performance of the nomogram was measured by Harrell’s C-index and calibration with 100 bootstrap samples (19).

### Correlation Between Adenosine-to-Inosine RNA Editing-Based Risk Score Sites and Host Gene Expression

Since the four AIRS sites are all located at the 3′-untranslated region (3′-UTR) of host genes, correlations between the editing level of each site and expression of host gene were analyzed by integrating TCGA-BRCA RNA-seq data with ATIRE profiling.

### Gene Set Enrichment Analysis

To determine the effect of AIRS sites on whole transcriptome expression, RNA-seq data were downloaded from TCGA. The difference in an expressional file between low and medium/high AIRS patients was assessed using the “limma” package in R software (version 4.0.1). Differences with adjusted *p*-value <0.05 and |log_2_(Fold change)| ≥ 1.5 were considered to be significant. Then, Gene Set Enrichment Analysis (GSEA) was performed to determine the Kyoto Encyclopedia of Genes and Genomes (KEGG) pathways (20).

### Statistical Analysis

In addition to the aforementioned statistical analysis, correlations between the editing level of ATIRE sites and the expression of host genes were tested by Spearman’s correlation test. All tests were two-sided and evaluated by GraphPad Prism 9.0. *p* < 0.05 was considered to be statistically significant.

## Results

### Baseline Clinicopathological Characteristics

The clinicopathological characteristics of all patients to be analyzed were presented in **Table 1**. There were no obvious differences in distributions of clinicopathological characteristics between the training and internal validation cohorts, but more cases in the external cohort were young and diagnosed at early stages with negative PR or positive HER2.

**Table 1:** Frequency distributions of clinicopathological features of BRCA cases.

Variables	Training set n (%)	Internal validation set n (%)	External validation set n (%)	*P* value *^a^*
Age at diagnosis				
<50 years	122 (27.0)	86 (27.7)	100 (56.8)	<0.001
≥50 years	330 (73.0)	225 (72.3)	76 (43.2)	
T stages				
Tris+1	131 (39.0)	77 (24.8)	93 (52.8)	<0.001
2	260 (57.5)	190 (61.1)	76 (43.2)	
3+4	61 (13.5)	44 (14.1)	7 (4.0)	
N stages				
0	230 (50.9)	150 (48.2)	89 (50.6)	0.774
1	141 (31.2)	111 (35.7)	57 (32.4)	
2+3	81 (17.9)	50 (16.1)	30 (17.0)	
M stages				
0	446 (98.7)	304 (97.7)	171 (97.2)	0.403
1	6 (1.3)	7 (2.3)	5 (2.8)	
Clinical stages				
I	90 (19.9)	48 (15.4)	61 (34.7)	<0.001
II	257 (56.9)	193 (62.1)	77 (43.7)	
III+IV	105 (23.2)	70 (22.5)	38 (21.6)	
ER status *^b^*				
Positive	331 (76.6)	239 (79.7)	128 (72.7)	0.219
Negative	101 (23.4)	61 (20.3)	48 (27.3)	
PR status *^b^*				
Positive	291 (67.4)	211 (70.3)	101 (57.4)	0.013
Negative	141 (32.6)	89 (29.7)	75 (42.6)	
HER2 *^b^*				
Positive	74 (24.0)	45 (21.7)	59 (33.5)	0.020
Negative	234 (76.0)	162 (78.3)	117 (66.5)	

BRCA, breast cancer; ER, estrogen receptor; PR, progesterone receptor; HER2, human epidermal growth factor receptor 2.

a P value from the chi-square test.

b Missing or ambiguous samples were omitted.

### Identification of Breast Cancer Survival-Related Adenosine-to-Inosine RNA Editing Sites

**Figure 2:**
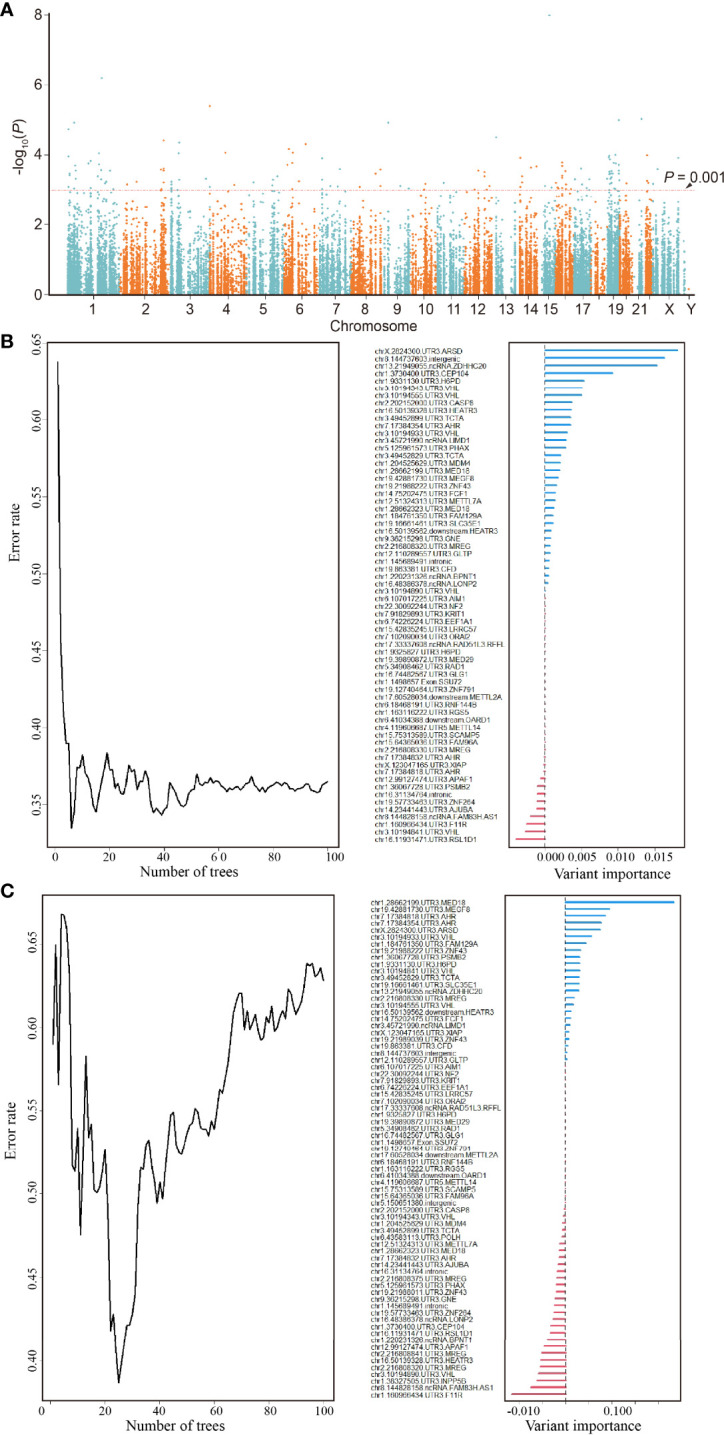
Identification of an ATIRE prognostic signature for OS and DFS of BRCA patients. **(A)** Scatter plot of *p*-values in −log10 scale from the univariate Cox-PH model on associations between all ATIRE sites and OS of TCGA-BRCA patients. y-Axis refers to *p*-values in –log10 scale, and x-axis refers to chromosomal location of these ATIRE sites. **(B)** Random forest modeling (left) and analysis of variable importance of ATIRE sites (right) to OS of BRCA. Left: survival forest of 100 trees was created, using the log-rank splitting rule with ATIRE predictors randomly selected at each split. Right: survival random forest analysis furnishes a ranking of ATIRE predictors’ importance in determining the accuracy of prediction. **(C)** Random forest modeling (left) and analysis of variable importance of ATIRE sites (right) to DFS of BRCA. Left: survival forest of 100 trees was created, using the log-rank splitting rule with ATIRE predictors randomly selected at each split. Right: survival random forest analysis furnishes a ranking of ATIRE predictors’ importance in determining the accuracy of prediction. ATIRE, adenosine-to-inosine RNA editing; OS, overall survival; DFS, disease-free survival; BRCA, breast cancer; TCGA, The Cancer Genome Atlas.

The Manhattan plot (**Figure 2A**) shows 137 ATIRE sites with *p* < 0.001 in the Cox-PH model for testing associations between 30,001 ATIRE sites and BRCA OS in the training cohort. Then the forest algorithm analysis featured 18 (**Figure 2B**) and 14 (**Figure 2C**) sites as foremost predictive factors for BRCA OS and DFS, of which seven sites, that are, ARSD A2874>I, ZNF791 A2280>I, H6PD A8760>I, MED18 A1552>I, MEGF8 A9749>I, SSU27 A1727>I, and RAD1 A1415>I, were overlapped. The number residing in A>I indicates the distance between the transcription start site and the ATIRE site.

**Figure 3:**
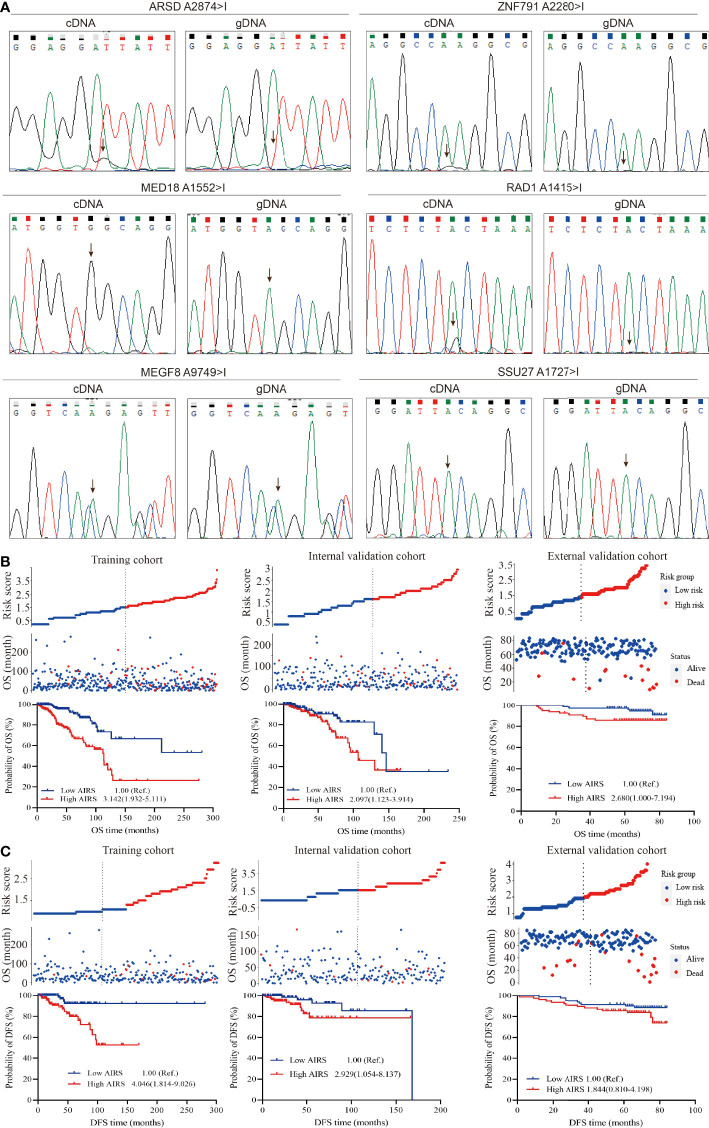
Correlation between AIRS, OS, and DFS of BRCA patients. **(A)** Validation of the selected ATIRE sites by direct sequencing. cDNA, complementary DNA; gDNA, genomic DNA. **(B)** Distribution of the risk score (top), survival status (middle), and Kaplan–Meier plot (bottom) to visualize the OS probabilities for AIRS in the training cohort (left), internal validation cohort (middle), and external validation cohort (right). **(C)** Distribution of the risk score (top), survival status (middle), and Kaplan–Meier plot (bottom) to visualize the DFS probabilities for AIRS in the training cohort (left), internal validation cohort (middle), and external validation cohort (right). Hazard ratio (HR) values were calculated by the Cox regression model, as shown in the Kaplan–Meier plot. AIRS, adenosine-to-inosine RNA editing-based risk score; OS, overall survival; DFS, disease-free survival; BRCA, breast cancer; ATIRE, adenosine-to-inosine RNA editing.

Direct sequencing (**Figure 3A**) shows ARSD A2874>I, ZNF791 A2280>I, MED18 A1552>I, and RAD1 A1415>I with distinct G peak in the cDNA but not in the corresponding gDNA, suggesting that these sites were edited. However, no edit of MEGF8 A9749>I and SSU27 A1727>I were observed. Besides, the trace peaks for H6PD were misshapen, causing a failed determination of H6PD A8760>I. Thus, the four experimentally confirmed ATIRE sites were selected to construct the AIRS model for BRCA OS and DFS.

### Adenosine-to-Inosine RNA Editing-Based Risk Score Construction and Its Association With Breast Cancer Overall Survival and Disease-Free Survival

After grouping the editing level of aforesaid screened ATIRE sites into low, medium, and high using the X-Tile Software, the HRs (**Supplementary Table S3**) for associations between these sites and BRCA OS were used to calculate AIRS. We generated a simple applet in Excel to easily calculate AIRS (**Supplementary File 1**). As shown in **Figure 3B**, after grouping AIRS by the median, we found that high AIRS trebled (HR = 3.142, 95%CI = 1.932–5.111) and doubled (HR = 2.097, 95%CI = 1.123–3.914) a BRCA patient’s risk of death in the training and internal validation cohorts. Being consistent, after removing 3 cases with undetermined editing levels in any of the AIRS sites, the high AIRS group displayed poorer OS than the low AIRS group (HR = 2.680, 95%CI = 1.000–7.194).

Similarly, the HRs for associations (**Supplementary Table S4**) between the aforesaid four sites and BRCA DFS were used to calculate AIRS. We also generated a simple applet in Excel to easily calculate AIRS for DFS (**Supplementary File 1**). As shown in **Figure 3C**, compared to the patients with low AIRS, those with high AIRS had a 4.046-fold (HR = 4.046, 95%CI = 1.814–9.026) and 2.929-fold (HR = 2.929, 95%CI = 1.054–8.137) risk of progression, recurrence, or death in the training and internal validation cohorts. In the external cohort, the high AIRS was associated with an increased risk (HR = 1.844, 95%CI = 0.810–4.198), but the association did not reach statistical significance, which may be due to the limited sample size. Taken together, these data suggest that AIRS is a prognostic indicator of BRCA OS and DFS.

### Adenosine-to-Inosine RNA Editing-Based Risk Score-Based Nomogram Demonstrated a Well Predictive Performance on Breast Cancer Survival

**Figure 4:**
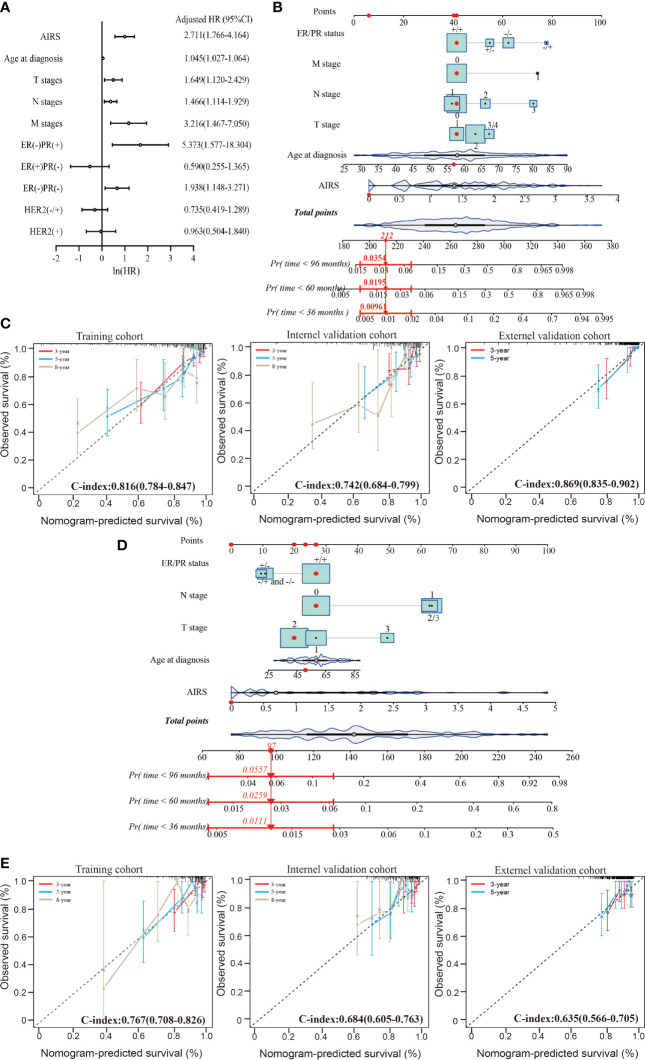
Performance of nomogram based on the AIRS and clinicopathological features. **(A)** Forest plot to visualize the HRs and 95%CIs for associations between the AIRS, clinicopathological features, and OS, after the multivariate Cox-PH model. **(B)** AIRS nomogram predicting 36-, 60-, and 96-month OS probability of BRCA patients. Each clinicopathological variable or AIRS has a certain number of the score (top row) ranging from 0 to 100. The sum of points of each variable was related to the probability of OS at specific time points (36, 60, and 96 months). The red dots and straight line with arrow show an example illustrating the use of the nomogram. This patient was one of TCGA-BRCA patients. The patient was ER^+^/PR^+^ (point = 41), no lymphatic metastasis (point = 41), no distant metastasis (point = 41), T1-stage (point = 41), 57-year-old (point = 42), and 0 ATRS (point = 6); thus, the total points of this patient is 212, which corresponds to a 99.9% probability of 36-month OS, a 98.1% probability of 60-month OS, and a 96.5% probability of 96-month OS. **(C)** The calibration curves for predicting patients’ OS at 3-year (red line), 5-year (violet-red line), and 8-year (tan line) in the training cohort (left), internal validation cohort (middle), and external validation cohort (right). **(D)** AIRS nomogram predicting 36-, 60-, and 96-month DFS probability of BRCA patient and an example illustrating the use of the nomogram. **(E)** The calibration curves for predicting patients’ DFS at 3-year (red line), 5-year (violet-red line), and 8-year (tan line) in the training cohort (left), internal validation cohort (middle), and external validation cohort (right). ER, estrogen receptor; PR, progesterone receptor; AIRS, adenosine-to-inosine RNA editing-based risk score; HRs, hazard ratios; OS, overall survival; TCGA, The Cancer Genome Atlas; BRCA, breast cancer.

The multivariate Cox-PH analysis showed that AIRS, ER/PR status, T stage, N stage, and M stage were independent predictors of mortality in TCGA patients affected by BRCA (**Figure 4A**). Thus, these factors were incorporated to develop a nomogram for predicting BRCA OS (**Figure 4B**). As shown in **Figure 4C**, the nomogram-predicted survival rate displayed a superior agreement with the observed survival rate in all the three cohorts, with Harrell’s C-indexes as 0.816 (95%CI = 0.784–0.847), 0.742 (95%CI = 0.684–0.799), and 0.869 (95%CI = 0.835–0.902).

The aforementioned prognostic factors were also used to develop a nomogram for predicting BRCA DFS based on the training cohort (**Figure 4D**). In this nomogram, the M stage was removed, because there was only one case with distant metastasis. As shown in **Figure 4E**, the nomogram-predicted survival rate displayed a noteworthy agreement with the observed survival rate in the training cohort and validation cohorts, with Harrell’s C-indexes as 0.767 (95%CI = 0.708–0.826), 0.684 (95%CI = 0.605–0.763), and 0.635 (95%CI = 0.566–0.705). These data suggest that AIRS-based nomograms could be used to predict BRCA OS and DFS.

### Effects of the Adenosine-to-Inosine RNA Editing-Based Risk Score Sites on Gene Expression Network

**Figure 5:**
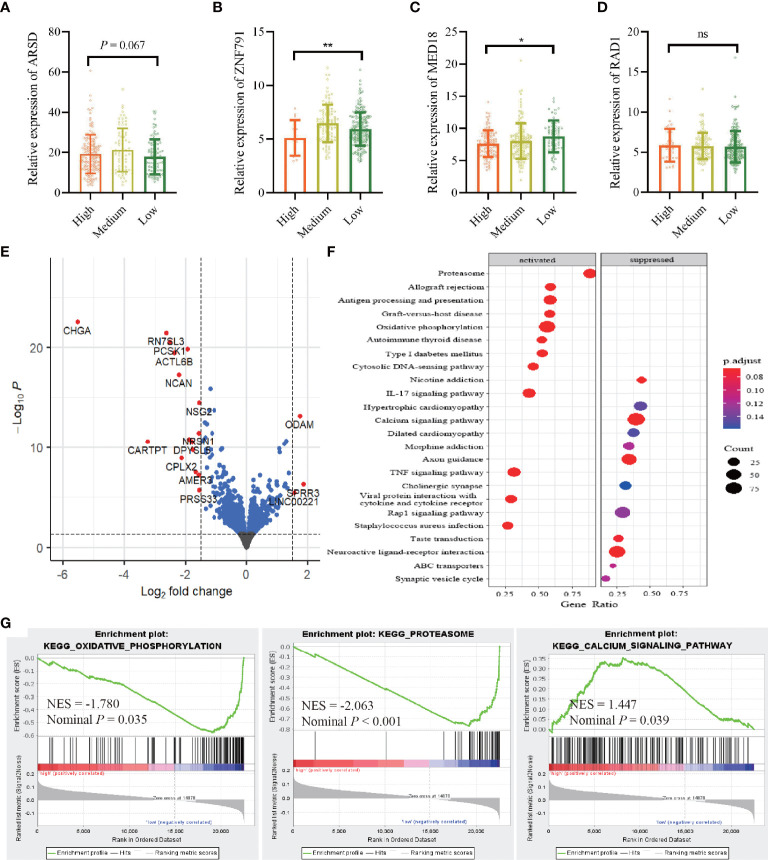
Correlation between the AIRS sites and gene expressions. **(A–D)** Correlations between editing levels of the four AIRS sites and expression levels of host genes. Data are presented as mean ± SD, and *p*-values are calculated by the one-way ANOVA test. *p < 0.05,**p < 0.01, ns, not significant. **(E)** Visualization of differentially expressed genes with volcano plot in high AIRS patients versus low AIRS patients. The expression difference of a log_2_ fold change of 1.5 (outer light gray broken vertical line) and for an adjusted *p*-value of 0.05 (dark broken horizontal line). y-Axis refers to *p*-values in–log10 scale, and x-axis refers to the fold change in log 2 scale. **(F)** Plot of the KEGG pathway enrichment analysis for AIRS-related genes based on TCGA data. y-Axis represents pathways; x-axis represents the amount of AIRS-related genes enriched in KEGG pathways. The color and size of each bubble represent enrichment significance and the number of AIRS-related genes enriched in KEGG, respectively. **(G)** GSEA enrichment plot of KEGG pathway genes in high AIRS versus low AIRS. KEGG, Kyoto Encyclopedia of Genes and Genomes; GSEA, Gene Set Enrichment Analysis; NES, normalized enrichment score; AIRS, adenosine-to-inosine RNA editing-based risk score; TCGA, The Cancer Genome Atlas.

Integrating TCGA-BRCA RNA-seq datasets with ATIRE profiling showed that a high editing level of A2280>I was significantly associated with a decreased level of ZNF791 when compared to a low or medium editing level (*p* = 0.002). Meanwhile, there was an inverse dose–effect relationship between the editing level of A1552>I and MED18 expression (*p* = 0.020). The editing level of A2874>I exerted a correlation with ARSD expression with a trend towards statistical significance (*p* = 0.067). However, no such effect was observed for A1415>I editing and RAD1 expression (**Figures 5A–D**). Moreover, a comparison of transcriptomic data between the high AIRS and low AIRS groups of TCGA-BRCA samples demonstrated that AIRS was correlated with expressions of 20 genes, of which 16 were lower and 4 were higher in the high AIRS group (**Figure 5E**; **Supplementary File 2**). KEGG pathways enrichment analysis showed that high AIRS actives several pathways such as oxidative phosphorylation and proteasome and suppresses pathways such as the calcium signaling pathway (**Figure 5F**). Consistently, GSEA with the 50 hallmarks found that signaling pathways of target genes are involved in oxidative phosphorylation, proteasome, and calcium signaling pathway (**Figure 5G**).

## Discussion

Although an overwhelming majority of studies have established many prognostic prediction models for BRCA *via* analyzing the transcriptomics data, most of these studies around this topic redundantly disaggregated transcriptomics data into gene sets by functional annotation to create new nomograms such as *N* ^6^-methyladenosine regulator-related or autophagy gene-related nomogram (21, 22). This excessive mining of transcriptomics actually contributes little to improve predictive precision of cancer prognosis, even if we combined these models considering the collinearity of gene expression. Besides, a lot of similar nomograms confuse the selection of such models for application. To overcome these problems, new developments in discovering novel biomarkers are needed. In this study, we for the first time constructed an AIRS model that exerts high associations with both OS and DFS of BRCA. We then developed a nomogram incorporating the AIRS and clinicopathological features, which performed precisely in predicting the survival of patients affected by BRCA.

Emerging evidence has demonstrated that dysregulated ATIRE was implicated in cancer development by causing amino acid changes, mRNA abundance and splicing anomaly, and re-direction of mRNA–microRNA binding (23, 24). The bioinformatics analyses on high-throughput RNA-seq data have widely revealed ATIRE sites and reported cancer survival-related ATIRE profiles, although these sites have not been actually verified (15, 16). Indeed, in this study, only four of the seven preselected sites were successfully verified by direct sequencing, demonstrating that identification of ATIRE sites *via* bioinformatics had some disadvantages of the uncertainties, with quite a deviation. Furthermore, multiple experiment studies targeting ATIRE in indicated genes or microRNAs have revealed several functional ATIRE sites to be associated with various cancer survival (25–32). These findings make it possible to use ATIRE sites as a potential tool in the assessment of cancer prognosis.

By mining TCGA-BRCA ATIRE profiling with a machine learning strategy, forest algorithm analysis, and by proving experimentation with direct sequencing, we for the first time identified the four ATIRE-relevant prognostic signatures and established the AIRS model for both OS and DFS of BRCA. It was found that the AIRS was associated with OS and DFS of BRCA in the internal and external validation cohorts, which agrees with the result from the training cohort. This indicates the validity of the model. The AIRS model was applied together with age at diagnosis, TNM stages, and ER and PR status to build a nomogram for predicting OS and DFS probability of patients affected by BRCA. Overall, our nomogram presents a good performance, robustness, and stability on predicting BRCA OS with Harrell’s C-indexes as 0.816, 0.742, and 0.869 in the three cohorts. The nomogram also exerts a considerable accuracy on predicting BRCA DFS with the C-indexes as 0.767, 0.684, and 0.635 in the three cohorts. The predictive efficiencies were also confirmed by the calibration plots. Thus, the nomogram may help the clinics for better health administration of patients with BRCA.

Since the four AIRS sites are all located in the 3′-UTR of host genes, it is plausible to observe the correlations between A2280>I editing level and ZNF791 expression, A1552>I editing level, and MED18 expression. However, the biological functions of ZNF791 and MED18 in BRCA are largely unknown, making it impossible to infer the plausibility of associations between the two sites and BRCA survival. Moreover, the cellular pathway clearly demonstrated that AIRS was associated with several signaling pathways, such as oxidative phosphorylation (33), proteasome (34), and calcium signaling pathway (35). These pathways are all promising targets for the treatment of BRCA, which suggests the AIRS as new targets for BRCA treatment.

One of the strengths of this study is that it represents a comprehensive examination of the whole transcriptomic ATIRE sites. The other one is that we used both internal and external validation cohorts to verify the AIRS model and nomogram. Nevertheless, there still remain some limitations in our research. First, functional analysis is lacking to support the biological plausibility of the association between the ATIRE sites and BRCA survival. Second, the omission of detailed treatment options in TCGA data prevents us to analyze the effect of the ATIES model on the effectiveness of different treatment strategies, which is essential in the future application of prognostic nomogram for treatment selection. Finally, bias, especially selection bias, may affect the authenticity of the association.

## Conclusion

The novel four-ATIRE signature serves as a promising model to predict the survival of patients with BRCA, and these ATIRE sites might be novel therapeutic targets for BRCA treatment, which is warranted to be further studied.

## Data Availability Statement

The original contributions presented in the study are included in the article/**Supplementary Material**. Further inquiries can be directed to the corresponding authors.

## Ethics Statement

The studies involving human participants were reviewed and approved by the Institutional Review Board of Guangzhou Medical University. The patients/participants provided their written informed consent to participate in this study.

## Author Contributions

Conceptualization: LY and QW. Data analysis: JW and SC. Sample resources: JW, AZ, YZ, QL, and YL. Data curation, LY. Experimental operation: ZY and YW. Writing—original draft preparation: JW. Writing—review and editing: LY and QW. Funding acquisition: LY. All authors have read and agreed to the published version of the manuscript.

## Funding

This research was funded by the National Natural Science Foundation of China grants, grant numbers 81871876, 82073628, and 81672303 (LY). The funders had no role in study design, data collection and analysis, decision to publish, or preparation of the manuscript.

## Conflict of Interest

The authors declare that the research was conducted in the absence of any commercial or financial relationships that could be construed as a potential conflict of interest.

## Publisher’s Note

All claims expressed in this article are solely those of the authors and do not necessarily represent those of their affiliated organizations, or those of the publisher, the editors and the reviewers. Any product that may be evaluated in this article, or claim that may be made by its manufacturer, is not guaranteed or endorsed by the publisher.
