# Supplementary material for: Development and Validation of a Four Adenosine-to-Inosine RNA Editing Site-Relevant Prognostic Signature for Assessing Survival in Breast Cancer Patients
Source: Front Oncol. 2022 Apr 12;12:861439. doi: 10.3389/fonc.2022.861439 (PMC9039306; doi:10.3389/fonc.2022.861439)
Supplement: Supplementary file 3 [file Table_1.docx]

**Supplementary Table S1.** Frequency distribution of clinic-pathological characteristics of BRCA cases in the external cohort.

| Variables | OS | | *P* value *^a^* |  | DFS | | *P* value *^a^* |
| --- | --- | --- | --- | --- | --- | --- | --- |
|  | Cases lost to follow-up  (n = 21) | Cases to be analyzed  (n = 176) |  |  | Cases lost to follow-up  (n = 32) | Cases to be analyzed  (n = 165) |  |
| Age at diagnosis |  |  |  |  |  |  |  |
| < 50 years | 11 (52.4) | 100 (56.8) | 0.698 |  | 14 (43.8) | 97 (58.8) | 0.116 |
| ≥ 50 years | 10 (47.6) | 76 (43.2) |  |  | 18 (56.2) | 68 (41.2) |  |
| T stages |  |  |  |  |  |  |  |
| Tris+1 | 7 (33.3%) | 93 (52.8%) | 0.236 |  | 13 (40.6%) | 87 (52.7%) | 0.388 |
| 2 | 13 (61.9%) | 76 (43.2%) |  |  | 18 (56.3%) | 71 (43.0%) |  |
| 3+4 | 1 (4.8%) | 7 (4.0%) |  |  | 1 (3.1%) | 7 (4.3%) |  |
| N stages |  |  |  |  |  |  |  |
| 0 | 8 (38.1%) | 89 (50.6%) | 0.375 |  | 16 (50.0%) | 81 (49.1%) | 0.986 |
| 1 | 7 (33.3%) | 57 (32.4%) |  |  | 10 (31.3%) | 54 (32.7%) |  |
| 2+3 | 6 (28.6%) | 30 (17.0%) |  |  | 6 (18.7%) | 30 (18.2%) |  |
| M stages |  |  |  |  |  |  |  |
| 0 | 21 (100.0%) | 171 (97.2%) | 0.434 |  | 32 (100.0%) | 160 (97.0%) | 0.319 |
| 1 | 0 (0.0%) | 5 (2.8%) |  |  | 0 (0.0%) | 5 (3.0%) |  |
| Clinical stages |  |  |  |  |  |  |  |
| I | 5 (23.8%) | 61 (34.7%) | 0.568 |  | 8 (25.0%) | 58 (35.2%) | 0.315 |
| II | 10 (47.6%) | 77 (43.7%) |  |  | 18 (56.3%) | 69 (41.8%) |  |
| III+IV | 6 (28.6%) | 38 (21.6%) |  |  | 6 (18.7%) | 38 (23.0%) |  |

*^a^ P* value from the chi-square test.

**Supplementary Table S2.** Primer information used for PCR and sequencing.

| Target gene | Forward sequence 5' → 3' | Reverse sequence5' → 3' |
| --- | --- | --- |
| ARSD A2874>I | TGAGCAACACAGTGAGAC | ACTTGGAAGAGAACACACC |
| ZNF791 A2280>I | AGTAGCAGTAGTAACACCATAG | GATCTCAGCTCACTGTCAG |
| MED18 A1552>I | GGATGTCACGGAGAATCTA | CCTCTAATGTTACCAACCTT |
| RAD1 A1415>I | GGCTTTAATCCCAGCACTT | GCAATGGCGTCATCTCAG |
| MEGF8 A9749>I | GGAAGCTGTTGGATGGTT | GGTTCAAGCGATTCTTCTG |
| SSU27 A1727>I | CTGTCATGCCTGGCTAAT | CCACTCCTGGCTACCTAA |
| H6PD A8760>I | ATGAGAGCCACCCATTGCC | CGCCTGTAATCCCAGCACTT |

**Supplementary Table S3.** HRs for associations between the selected ATIRE sites and BRCA OS were used to calculate AIRS.

| ATIRE site  (editing level) | n *^a^* | Death (%) | HR *^b^* | *P* value *^b^* |
| --- | --- | --- | --- | --- |
| ARSD A2874>I |  |  |  |  |
| High (>3%) | 195 | 18 (9.23) | 1.000(ref.) |  |
| Medium (2%-3%) | 100 | 20 (20.00) | 2.198(1.160-4.166) | 0.016 |
| Low (<2%) | 121 | 28 (23.14) | 3.076(1.699-5.570) | <0.001 |
| ZNF791 A2280>I |  |  |  |  |
| Low (0%) | 260 | 34 (13.08) | 1.000(ref.) |  |
| Medium (>0%-10%) | 142 | 25 (17.61) | 1.558(0.925-2.625) | 0.095 |
| High (>10%) | 14 | 7 (50.00) | 4.579(2.015-10.405) | <0.001 |
| MED18 A1552>I |  |  |  |  |
| High (>76%) | 137 | 15 (10.95) | 1.000(ref.) |  |
| Medium (55%-76%) | 202 | 35 (17.33) | 1.789(0.975-3.281) | 0.060 |
| Low (<55%) | 77 | 16 (20.78) | 2.392(1.178-4.856) | 0.016 |
| RAD1 A1415>I |  |  |  |  |
| Low (<1.8%) | 242 | 32 (13.22) | 1.000(ref.) |  |
| Medium (1.8%-6.0%) | 128 | 23 (17.97) | 1.655(0.965-2.838) | 0.067 |
| High (>6.0%) | 46 | 11 (23.91) | 2.597(1.298-5.198) | 0.007 |

*^a^* Samples with undetermined editing level of the site were omitted.

*^b^* HR and *P* value from a Cox-PH regression test.

**Supplementary Table S4.** HRs for associations between the selected ATIRE sites and BRCA DFS were used to calculate AIRS.

| ATIRE site  (editing level) | n *^a^* | Death (%) | HR *^b^* | *P* value *^b^* |
| --- | --- | --- | --- | --- |
| ARSD A2874>I |  |  |  |  |
| High (>2.6%) | 145 | 7 (4.83) | 1.000(ref.) |  |
| Medium (1.2%-2.6%) | 76 | 10 (13.16) | 2.479(0.941-6.535) | 0.066 |
| Low (<1.2%) | 41 | 7 (17.07) | 4.877(1.705-13.948) | 0.003 |
| ZNF791 A2280>I |  |  |  |  |
| Low (0%) | 174 | 12 (6.90) | 1.000(ref.) |  |
| High (>0%) | 88 | 12 (13.64) | 2.244(1.007-5.001) | 0.048 |
| MED18 A1552>I |  |  |  |  |
| High (>76%) | 87 | 7 (8.05) | 1.000(ref.) |  |
| Medium (52%-76%) | 139 | 11 (7.91) | 1.337(0.516-3.466) | 0.516 |
| Low (<52%) | 36 | 6 (16.67) | 3.067(1.024-9.187) | 0.045 |
| RAD1 A1415>I |  |  |  |  |
| Low (<1.8%) | 152 | 13 (8.55) | 1.000(ref.) |  |
| Medium (1.8%-6.0%) | 79 | 5 (6.33) | 1.204(0.425-3.416) | 0.726 |
| High (>6.0%) | 31 | 6 (29.35) | 3.911(1.469-10.413) | 0.006 |

*^a^* Samples with undetermined editing level of the site were omitted.

*^b^* HR and *P* value from a Cox-PH regression test.
